# Rationale and design of the Multidisciplinary Approach to Novel Therapies in Cardiology Oncology Research Trial (MANTICORE 101 - Breast): a randomized, placebo-controlled trial to determine if conventional heart failure pharmacotherapy can prevent trastuzumab-mediated left ventricular remodeling among patients with HER2+ early breast cancer using cardiac MRI

**DOI:** 10.1186/1471-2407-11-318

**Published:** 2011-07-27

**Authors:** Edith Pituskin, Mark Haykowsky, John R Mackey, Richard B Thompson, Justin Ezekowitz, Sheri Koshman, Gavin Oudit, Kelvin Chow, Joseph J Pagano, Ian Paterson

**Affiliations:** 1 University of Alberta, Edmonton, Alberta, Canada; 2 Cross Cancer Institute, Edmonton, Alberta, Canada; 3 Department of Cardiology, Walter McKenzie Center, Edmonton, Alberta, Canada

**Keywords:** Cardiotoxicity, Heart failure, Cardiac remodeling, Trastuzumab, Breast cancer

## Abstract

**Background:**

MANTICORE 101 - Breast (Multidisciplinary Approach to Novel Therapies in Cardiology Oncology Research) is a randomized trial to determine if conventional heart failure pharmacotherapy (angiotensin converting enzyme inhibitor or beta-blocker) can prevent trastuzumab-mediated left ventricular remodeling, measured with cardiac MRI, among patients with HER2+ early breast cancer.

**Methods/Design:**

One hundred and fifty-nine patients with histologically confirmed HER2+ breast cancer will be enrolled in a parallel 3-arm, randomized, placebo controlled, double-blind design. After baseline assessments, participants will be randomized in a 1:1:1 ratio to an angiotensin-converting enzyme inhibitor (perindopril), beta-blocker (bisoprolol), or placebo. Participants will receive drug or placebo for 1 year beginning 7 days before trastuzumab therapy. Dosages for all groups will be systematically up-titrated, as tolerated, at 1 week intervals for a total of 3 weeks. The primary objective of this randomized clinical trial is to determine if conventional heart failure pharmacotherapy can prevent trastuzumab-mediated left ventricular remodeling among patients with HER2+ early breast cancer, as measured by 12 month change in left ventricular end-diastolic volume using cardiac MRI. Secondary objectives include 1) determine the evolution of left ventricular remodeling on cardiac MRI in patients with HER2+ early breast cancer, 2) understand the mechanism of trastuzumab mediated cardiac toxicity by assessing for the presence of myocardial injury and apoptosis on serum biomarkers and cardiac MRI, and 3) correlate cardiac biomarkers of myocyte injury and extra-cellular matrix remodeling with left ventricular remodeling on cardiac MRI in patients with HER2+ early breast cancer.

**Discussion:**

Cardiac toxicity as a result of cancer therapies is now recognized as a significant health problem of increasing prevalence. To our knowledge, MANTICORE will be the first randomized trial testing proven heart failure pharmacotherapy in the prevention of trastuzumab-mediated cardiotoxicity. We expect the findings of this trial to provide important evidence in the development of guidelines for preventive therapy.

**Trial Registration:**

ClinicalTrials.gov: NCT01016886

## Background

Breast cancer is the most common malignancy and second leading cause of cancer death [1]. Approximately 20-25% of breast cancers over-express human epidermal growth factor receptor 2 (HER2+) which is associated with poor prognosis [2,3]. Trastuzumab (Herceptin^®^), a humanized monoclonal antibody targeting the HER2 receptor, was previously shown to improve survival by 20% in women with HER2+metastatic disease [4-7]. More recently, 4 major adjuvant trials of women with HER2+ early breast cancer (EBC) demonstrated that trastuzumab reduced 3-year breast cancer recurrence and risk of death rate by 50% [8]. Given these positive findings, trastuzumab was approved in 2006 by the Food and Drug Administration for the adjuvant treatment of HER2+ breast cancer.

Despite favourable survival benefits, an adverse effect of trastuzumab is (a)symptomatic left ventricular (LV) dysfunction and heart failure (HF). In the phase III trials, HF and asymptomatic LV dysfunction was reported in 4% and 18% of patients, respectively [9-11]. Although trastuzumab-related cardiotoxicity has been considered 'reversible' [12], Wadhwa et al. reported that trastuzumab was stopped in 22% of patients due to asymptomatic LV systolic dysfunction; notably, of these, 40% showed no improvement or worsening of LV function over time despite optimal pharmacotherapy [13]. Similarly, Chia observed that 21.6% of women receiving adjuvant trastuzumab-based chemotherapy experienced a cardiac event requiring temporary or permanent discontinuation of trastuzumab [14]. These observations are important, given the influence of more stringent cardiac exclusion criteria in the pivotal trials compared to standard clinical practice. Furthermore, any dose reductions, delay or discontinuation due to cardiotoxicity are potentially life-threatening events from the competing risks of cancer and/or cardiac mortality. Therefore, better understanding of the pathophysiology of trastuzumab-mediated cardiotoxicity and its prevention are urgently required.

Ventricular remodeling (increased cavity size and decreased pump function) precedes overt HF [15-17]. Our group has shown that aerobic exercise training has beneficial anti-remodeling benefits in clinically stable systolic HF patients [18]. Based on these findings, we examined the effect of 4 months of aerobic exercise training on LV remodeling in 17 women with EBC receiving trastuzumab-based chemotherapy [19]. We found that LV remodeling occurs early, confirming observations of other groups [14,20], and that an early exercise intervention did not attenuate remodeling in this setting. Accordingly, we identified the need for examination of non-exercise interventions.

Pharmacotherapy has been shown to attenuate or reverse LV remodeling in the HF and post-myocardial infarction (MI) setting. Angiotensin-converting enzyme inhibitors (ACEI) have been proven to delay or reverse LV dilation and improve ejection fraction (EF) in multiple trials [21-24]. Beta-blockers (BB) have also been shown to be beneficial, but have largely been tested in combination with other therapies [25,26]. To date, a paucity of studies have examined conventional HF therapy during anthracycline therapy [27-29]. Specifically, carvedilol has been shown to be an effective single-agent therapy in anthracycline-induced cardiomyopathy [30].Cardinale *et al *demonstrated that an ACEI can prevent a decline in EF and cardiac events in cancer patients receiving high dose anthracyclines [31]. In general, however, preventive medical therapy is not considered necessary with anthracycline-based regimens, as toxicity is related to the cumulative dose (> 500 mg/m^2 )^ [32].

Detection and measurement of LV dysfunction may be hampered by insensitivity of routinely-available imaging modalities. Most EBC clinical trials have employed either radionuclide ventriculography (eg. MUGA) or transthoracic echocardiograms (ECHO) which may underestimate LV volumes [33]. Cardiac MRI is the preferred method for the quantification of ventricular volumes and EF in individuals with impaired LV systolic function [34-36]. Unfortunately, relying on a decline in EF or overt clinical symptoms limits detection of cardiotoxicity to the late stage of disease process, increasing the likelihood of irreversible myocardial damage.

In addition to detailed imaging, cardiac biomarkers may contribute to early detection, assessment and longer-term monitoring of cardiotoxicity. In patients with multiple types of advanced cancer receiving high-dose chemotherapy, Cardinale and associates demonstrated that troponin I increased in one-third of patients shortly after treatment, which was associated with a concomitant reduction in EF within the subsequent year [31]. In serial evaluation of adjuvant and metastatic patients receiving trastuzumab-based chemotherapy, elevations in troponin were seen early in therapy (after 2 cycles) and with adjustment for major confounders, was the strongest independent predictor of future EF decline, occurring within 1 - 8 months [37]. In these patients (n = 42), a three-fold decrease in likelihood of recovery was observed over time despite optimal pharmacotherapy. Brain natriuretic peptide (BNP), an established marker of heart failure, has also been shown to predict cardiac events including symptomatic heart failure, arrhythmias and acute coronary syndrome in patients receiving anthracycline-based chemotherapy [38]. While these studies provide valuable insights, larger studies in homogeneous patient groups with similar chemotherapy regimens and biomarker collection are required before general recommendations can be implemented [39]. Finally, other novel markers may have the potential to detect early cardiac dysfunction. For example, collagen-derived peptides have been correlated to the fractional volume of myocardial fibrosis in hypertensive patients [40]and HF[41]. As the underlying collagen-based structure of the heart remodels, peptides are released into the plasma [42-44]. While these molecules have not been studied in cancer patients [45], prospective exploration of these markers and other prospectively-collected biofluids may provide insight into disease evolution or effects of pharmacotherapy.

To date, no study has prospectively examined the effects of proven HF therapy to prevent cardiotoxicity in patients with EBC receiving trastuzumab. The median follow-up of the pivotal trastuzumab trials remains between two and three years, limiting understanding of the clinical course of patients with trastuzumab mediated cardiac toxicity and thus, the appropriate length of therapy. Therefore, the logical extension of our body of research is to examine: (1) the underlying pathogenesis of trastuzumab mediated cardiac toxicity, (2) screening tools for detecting early LV remodeling in this patient group, (3) determine the evolution of LV remodeling beyond chemotherapy, and (4) pharmacotherapies to prevent LV remodeling for these patients.

Given this background, our cardio-oncology research group designed the *Multidisciplinary Approach to Novel Therapies In Cardiology Oncology Research (MANTICORE) trial. *The primary objective of this randomized clinical trial is to determine if conventional heart failure pharmacotherapy (ACEI or BB) can prevent trastuzumab-mediated LV remodeling among patients with HER2+ EBC, determined by 12 month change in LV end-diastolic volume (LVEDV) measured using cardiac MRI. Secondary objectives include 1) determine the evolution of LV remodeling on cardiac MRI in patients with HER2+ EBC; 2) understand the mechanism of trastuzumab mediated cardiac toxicity by assessing for the presence of myocardial injury and apoptosis using serum biomarkers and cardiac MRI; and 3) correlate cardiac biomarkers of myocyte injury and extra-cellular matrix remodeling with LV remodeling on cardiac MRI.

## Methods/Design

This study is parallel 3-arm, randomized, placebo controlled, double-blind study comparing bisoprolol and perindopril for the prevention of LV remodeling in patients with HER2+ breast cancer treated with adjuvant trastuzumab. Additional inclusion requirements are adequate creatinine clearance, age > 18 years, no contraindication to MRI and willingness to provide informed consent. Exclusion criteria are known contraindication or current treatment with ACEI or BB; history of heart failure, cardiomyopathy, baseline EF < 50%; and history of uncontrolled hypertension or myocardial infarction. Ethical approval has been secured from the two relevant institutional review Boards.

### Participants and Setting

Potential participants are consecutively identified and screened for eligibility by the study coordinator during multidisciplinary Tumor Board review of all new breast cancer case consultations. Following primary oncologist approval, potential participants are provided with a review of the study and provided with written study information. After obtaining written consent, participants will be scheduled for a baseline clinic visit for final determination of eligibility.

### Group Allocation

**Figure 1:**
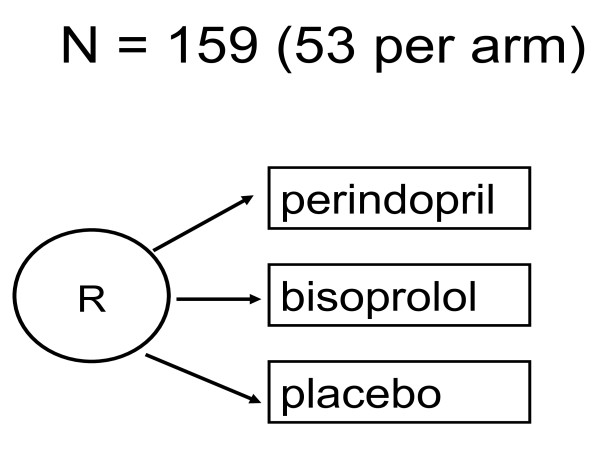
**Randomization **Randomization Scheme Diagram

Participants will be randomized in a 1:1:1 ratio to perindopril, bisoprolol, or placebo (Figure 1) using a secure internet randomization service (EPICORE, http://www.epicore.ualberta.ca).

### Pharmacotherapy

Participants will receive drug or placebo for 1 year beginning 7 days before trastuzumab therapy. There will be no run-in period. Dosages for all groups will be systematically up-titrated as tolerated at 1 week intervals, for a total of 3 weeks. Perindopril will be initiated at 2 mg and titrated to a target dose of 8 mg. Bisoprolol will be initiated at 2.5 mg and titrated to a 10 mg target dose. All dosages will be labeled by dose level (1, 2 or 3) and will be otherwise identical in appearance.

### Blinding and Masking

The study coordinator, investigators, treating oncologists and patient will all be blinded to drug allocation and outcomes. All cardiac MRIs will be read by a core lab at the Mazankowski Alberta Heart Institute (MAHI) in Edmonton, Canada, with 2 readers, blinded to treatment allocation. The treating oncologist will be provided with global EF measurement only (per standard of care).

### Evaluations

**Figure 2:**
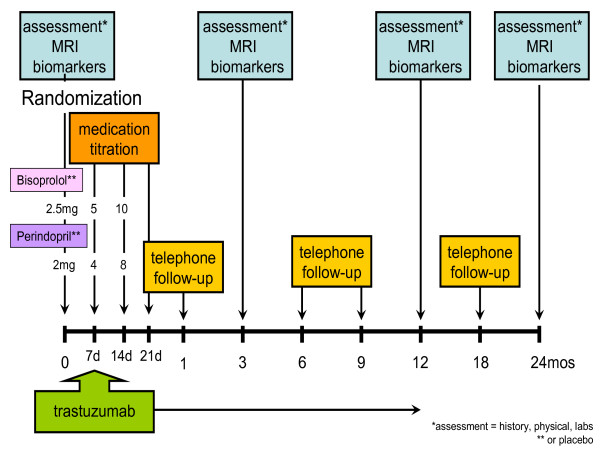
**Study Procedures **Flow Diagram of Study Procedures

Baseline demographics, medical history, cardiac risk factors, and cardiac history are evaluated. Physical examination including cardiovascular exam, blood pressure and pulse is performed. Blood is collected for the purposes of measuring baseline electrolytes, creatinine (for estimation of glomerular filtration rate [GFR]), and biomarkers (Figure 2) and an electrocardiogram. Participants undergo a comprehensive baseline cardiac MRI as part of their routine pre-chemotherapy ventricular function evaluation in lieu of conventional modalities. An additional consent is obtained for prospective serial collection of serum and urine in the Alberta Research Tumor Bank (http://www.abtumorbank.com) for future analysis.

Participants initiate study drug one week prior to starting trastuzumab therapy and are scheduled for titration visits (Figure 2). Participants return for a visit with the study coordinator at 3 and 12 months after randomization to be assessed for signs and symptoms of HF, major adverse cardiac events (MACE), adverse effects relating to HF pharmacotherapy as well as adherence to medication. In addition, participants will be contacted by telephone at months 1, 6, 9 and 18 to ensure continued stability and minimize loss to follow-up. Participants will also undergo blood work to assess electrolytes and creatinine at 3 month intervals as per routine care while on trastuzumab therapy and have biomarkers drawn at 3 and 12 months for comparison with the concurrent cardiac MRI measures. Cardiac monitoring at 6 and 9 month intervals will be performed with routinely available modalities per standard of care. If trastuzumab therapy is discontinued due to cardiac toxicity outside of the 3 or 12 month cardiac MRI scans then participants will return for another clinically indicated cardiac MRI within 1 week of chemotherapy termination.

Participants will return for a final visit with the study coordinator at 24 months, or 1 year after trastuzumab therapy termination. The study coordinator will perform the same clinical assessment as on previous follow-up visits at 3 and 12 months. Evaluation of medications will be done to ascertain if treatment for HF has been initiated since the 12 month visit. Patients will undergo the same collection of serum biomarkers. A non-clinically indicated research cardiac MRI will be performed to assess for the presence of ongoing LV remodeling. The long-term cardiac implications of trastuzumab therapy are unknown but we hypothesize that the exposed heart will be more vulnerable to future insults [49].

### Data Security

De-identifying case report form development, data entry, quality assurance and data analysis will be performed by EPICORE. De-identified image data from cardiac MRIs will be stored on a secure network using unique identifiers. Blood samples for biomarker analysis will also only be identified by unique identifiers and stored for batch analysis.

## Statistical Considerations

### Outcome analyses

Participants will be analyzed with the intention to treat principle. The primary outcome analysis will be the difference in mean change in LVEDV at 1 year in each active treatment arm and the placebo arm, evaluated using a general linear model controlling for differences in patient characteristics at the baseline and 12 month assessments. A general linear mixed model will be used to assess group differences with serial correlation over time based on the baseline, 3, 12, and 24 month measurements with correlated post-hoc analysis where appropriate. All statistical tests will be two-sided. Regression analyses and diagnostic tests will be performed to model changes in continuous variables from imaging with those from biomarker measures. Statistical significance will be assumed at the 5% level (p < 0.05). Missing data will be interpolated where possible using multivariable imputation. Data will be analyzed using SAS 9.2 (SAS, Cary, NC) software.

### Sample size calculations

Based on our pilot data [19], we expect the placebo group will have a minimum change in LVEDV of +11% with 1-year of trastuzumab therapy. We assume that pharmacotherapy with ACEI would prevent 90% of the trastuzumab mediated remodeling as measured by a change in LVEDV at 1 year = +1%. As previously mentioned, ACEI and perindopril have been shown to entirely prevent LV remodeling in other models of heart failure. Note that the change in LV volume in our pilot study was observed after 4 months of trastuzumab; hence we are possibly underestimating the true magnitude of change in LV volume at 1 year in the placebo group of this study. This is supported by an interim analysis of the BCIRG 006 trial, showing that mean LV function tended to decrease out to 8 months regardless of the chemotherapy protocol [11]. If the within-group standard deviation is 20 ml, with a two-2-tailed significance level ∝ = 0.05 and a power of .80, then 47 participants are required for each group. We anticipate a 10% drop-out rate and a 3% mortality rate during our 2 year follow-up [46], therefore we will recruit a total 159 participants. If the absolute difference between the mean LVEDV in placebo and ACE inhibitor groups is greater than 10% at 1 year, this sample size will still be sufficiently powered to detect a lower degree of remodeling prevention (Table 1).

**Table 1:** Sample size calculations and magnitude of difference in left ventricular remodeling between treatment groups

**Placebo** **Δ LVEDV** **at 1 year**	**Perindopril** **Δ LVEDV** **at 1 year**	**% Remodeling prevented**
+11%	+1%	90%
+11.7%	+1.9%	84%
+12.5%	+2.75%	78%
+14.2%	+4.4%	69%
+15.8%	+6.1%	62%

### Tracking and Monitoring of Adverse Events

Participants in this trial will be exposed to the known risks of HF therapy however, follow-up visits, blood-work and phone calls will screen for potential adverse reaction. Cardiac MRI is a safe imaging modality with no radiation exposure to patients. Gadolinium contrast agents are now recognized to be associated with a rare life threatening condition called nephrogenic systemic fibrosis (NSF) however this complication is confined to those with GFR < 30 ml/min, an exclusion criterion of our study.

EPICORE will oversee an independent data safety and monitoring committee that will meet at quarterly intervals and review MACE to ensure that continuation of the study is safe. In the event that the participant will not be able to continue either the study drug or placebo, they will remain in their randomized group to preserve the intention-to-treat principle. Indications for open label therapy ACEI or BB therapy will include: (1) an absolute decline in EF by ≥ 5% and EF < 55% with symptoms of HF or (2) an asymptomatic absolute decline in EF by ≥ 10% and EF < 50%. Participants are free to withdraw at any time but will be invited to continue in their treatment arm for the duration of the study.

## Discussion

### Methodological Considerations

Given our small sample size, we do not expect to find a significant decrease in cardiac clinical outcomes, however, given the overwhelming evidence of clinical efficacy in the HF literature, we expect to demonstrate a beneficial effect of pharmacotherapy in preventing trastuzumab-mediated LV remodeling. Our results would then justify a multicenter trial studying the role of ACEI and/or BB for preventing clinical HF and improving long-term outcomes of breast cancer survivors. Furthermore, if ACEI or BB are proven efficacious in the prevention of cardiac toxicity, then patients with known cardiac dysfunction (thus ineligible to receive trastuzumab) could also be studied. Effective strategies for preventing trastuzumab mediated cardiac toxicity would allow more HER2+ breast cancer patients to successfully complete their adjuvant chemotherapy, while avoiding delays or discontinuation of vital therapy.

Our secondary objectives may also yield information on appropriate biomarkers to screen for cardiac toxicity and provide additional information on the underlying pathogenesis and the evolution of cardiac injury beyond exposure to trastuzumab. Ideal biomarkers in this field have yet to be identified, however, cardiac MRI allows for precise characterization of LV dysfunction against which biomarkers can be evaluated. In future studies, we will analyze prospectively collected biofluids in the study of metabolics and genomics of trastuzumab-related cardiotoxicity. Urine metabolomics have the capacity to sensitively measure metabolic processes at the cellular level, and may provide early insights into myocyte structural or energetic changes [47,48]. Molecular profiling may identify key pathways involved in tissue regeneration, drug metabolism and cell death [49,50]; in studying copy number variants, we have shown that toxicities associated with docetaxel-containing chemotherapy regimens may be predicted [51]. Taken together, study of biomarkers and clinical outcomes in this setting will contribute to personalized care and improved therapeutic outcomes.

## Conclusion

Cardiac toxicity as a result of cancer therapies is now recognized as a significant health problem of increasing prevalence. While therapies such as trastuzumab potentially offer improved survival with targeted anti-tumor activities, the additional cardiac insult in addition to 'multiple-hit' [52] injuries put patients at risk of morbidity and potentially premature mortality. A limitation of the existing data is that the median follow-up in the trastuzumab adjuvant trials is between 2 and 3 years. Therefore, information is lacking on the potential for late cardiac dysfunction, or whether short-term improvements in LV remodeling or EF with medical treatment are permanent or temporary. Future trials with longer follow-up are necessary to address these remaining issues.

To our knowledge, MANTICORE will be the first randomized trial testing proven HF pharmacotherapy in the prevention of trastuzumab-mediated cardiotoxicity. We expect the findings of this trial to provide high-level evidence in the development of guidelines for preventive therapy. Relationships of established and novel biomarkers to sensitive MRI measures will enhance our understanding of these markers, as well as development of sensitive assays for the earliest detection of cardiovascular changes in these patients. Lastly, this work will inform and support other long-term goals of our team, in the prevention, detection and treatment of cardiovascular effects of cancer therapies.

## Abbreviations

ACEI: angiotensin-converting enzyme inhibitor; BB: beta-blocker; BNP: brain natriuretic peptide; EBC: early breast cancer; ECHO: trans-thoracic echocardiogram; EF: ejection fraction; GFR: glomerular filtration rate; HER2: human epidermal growth factor receptor 2; HF: heart failure; LV: left ventricle; LVEDV: left ventricular end-diastolic volume; MACE: major adverse cardiac event; MAHI: Mazankowski Alberta Heart Institute; MRI: magnetic resonance imaging; MUGA: radionuclide ventriculography.

## Competing interests

There is no competing interest to declare on the part of any named author.

## Authors' contributions

EP: study concept and design, project coordination, manuscript development and revision; MH: study concept and design, manuscript review and revision; JM: study concept and design, manuscript review;

RT: study concept and design, MRI analysis and interpretation, manuscript review; JE: study concept and design, manuscript review and revision; SK: study concept and design, manuscript review and revision; GO: study concept and design, manuscript review and revision; KC: MRI data acquisition, analysis and interpretation; manuscript review; JP: MRI data acquisition, analysis and interpretation; manuscript review; IP: principal investigator, study concept and design, manuscript review and revision.

All authors have read and approved the final version of this manuscript.

## Pre-publication history

The pre-publication history for this paper can be accessed here:

http://www.biomedcentral.com/1471-2407/11/318/prepub
